# *OpenMMDL* - Simplifying the Complex: Building, Simulating, and Analyzing Protein–Ligand Systems in *OpenMM*

**DOI:** 10.1021/acs.jcim.4c02158

**Published:** 2025-02-11

**Authors:** Valerij Talagayev, Yu Chen, Niklas Piet Doering, Leon Obendorf, Katrin Denzinger, Kristina Puls, Kevin Lam, Sijie Liu, Clemens Alexander Wolf, Theresa Noonan, Marko Breznik, Petra Knaus, Gerhard Wolber

**Affiliations:** ‡ Department of Biology, Chemistry and Pharmacy, Institute of Pharmacy, Molecular Design Group, Königin-Luisestr. 2 + 4, 14195 Berlin, Germany; ¶ Department of Biology, Chemistry and Pharmacy, Institute of Biochemistry, Signal Transduction Group, Thielallee 64, 14195 Berlin, Germany

## Abstract

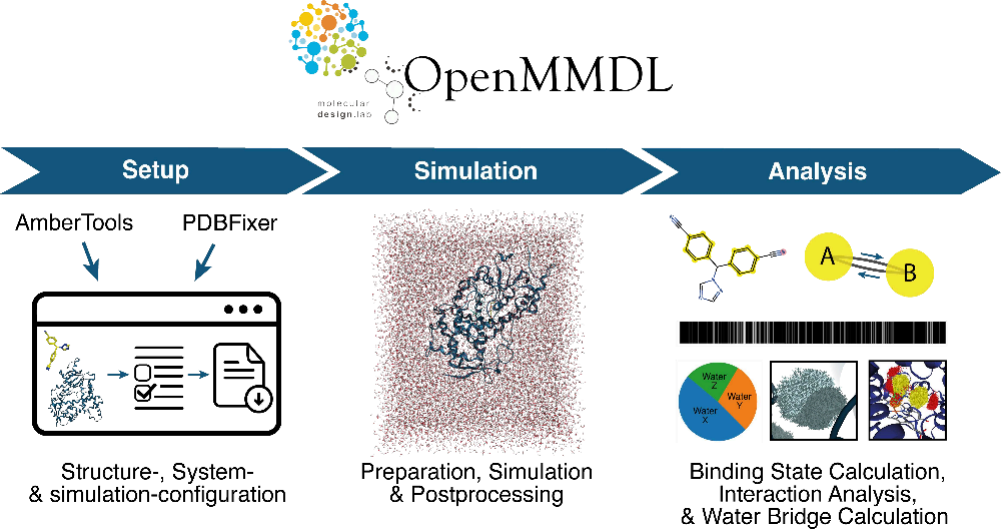

Molecular dynamics (MD) simulations have become an essential tool for studying the dynamics of biological systems and exploring protein–ligand interactions. *OpenMM* is a modern, open-source software toolkit designed for MD simulations. Until now, it has lacked a module dedicated to building receptor–ligand systems, which is highly useful for investigating protein–ligand interactions for drug discovery. We therefore introduce *OpenMMDL*, an open-source toolkit that enables the preparation and simulation of protein–ligand complexes in *OpenMM*, along with the subsequent analysis of protein–ligand interactions. *OpenMMDL* consists of three main components: *OpenMMDL Setup*, a graphical user interface based on Python *Flask* to prepare protein and simulation settings, *OpenMMDL Simulation* to perform MD simulations with consecutive trajectory postprocessing, and finally *OpenMMDL Analysis* to analyze simulation results with respect to ligand binding. *OpenMMDL* is not only a versatile tool for analyzing protein–ligand interactions and generating ligand binding modes throughout simulations; it also tracks and clusters water molecules, particularly those exhibiting minimal displacement from their previous coordinates, providing insights into solvent dynamics. We applied *OpenMMDL* to study ligand–receptor interactions across diverse biological systems, including LDN-193189 and LDN-212854 with ALK2 (kinases), nifedipine and amlodipine in Ca_*v*_1.1 (ion channels), LSD in 5-HT_2B_ (G-protein coupled receptors), letrozole in CYP19A1 (cytochrome P450 oxygenases), flavin mononucleotide binding the FMN-riboswitch (RNAs), ligand C08 bound to TLR8 (toll-like receptor), and PZM21 bound to MOR (opioid receptor), highlighting distinct functionalities of *OpenMMDL*. *OpenMMDL* is publicly available at https://github.com/wolberlab/OpenMMDL.

## Introduction

Molecular dynamics (MD) simulations have become an essential tool for conformational sampling of biological systems,^1^ protein–protein interactions^2^ and dynamics of protein–ligand interactions.^3^ Various MD software packages, including *Amber*,^4^*Gromacs*,^5^*CHARMM*,^6^*NAMD*,^7^ and *Desmond*(8) have been developed, each with unique benefits and drawbacks. Though these packages offer diverse features, they face extensibility challenges, with new methods often delayed by developers favoring specific tools. To address this, *OpenMM*(9) was developed as a community-driven tool, with customizable force fields, Python integration, and GPU acceleration. However, it lacks built-in easy preparation of protein–ligand simulations, limiting its use in drug discovery. To address this issue, users must rely on tools from third-party packages^4,10^ for ligand parametrization and wrapping. Despite the availability of helpful tutorials such as *TeachOpenCADD*,^11^ setting up receptor–ligand simulations remains complex, especially for noncoders or those with less MD experience. The development of user-friendly solutions was inspired by the need to simplify complex computational pipelines for broader accessibility. Our focus was on using and extending *OpenMM Setup*,^12^ a graphical user interface (GUI) developed by *OpenMM* developers.

Among the tools available for analyzing MD simulations,^13,14^ Python-based libraries such as *MDAnalysis*,^15^*MDTraj*,^16^ and *HTMD*(17) stand out for their seamless integration with Python-based simulation tools, like *OpenMM*.^9^ However, these libraries often lack a focus on protein–ligand interactions, a critical area for many studies. In order to address this, GUI-based tools like *OpenMMDL* require the integration of additional packages like the Protein–Ligand Interaction Profiler (*PLIP*),^18,19^ which analyzes protein–ligand complexes, defining interactions based on functional groups, distances, and angles.

Although the stability of ligand-protein interactions is often assessed by tracking their movements in MD trajectories, time-resolved, interaction-based analyses offer novel insights.^20−22^ Tools such as *Dynophores*(20−25) provide such analyses by applying abstraction layers to *LigandScout*’s^26^ interaction calculations. This approach considers atomic hybridization alongside geometrical features essential for chemical interactions. Another method, Protein–Ligand Interaction Fingerprints (*ProLIF*),^27^ uses SMARTS patterns^28^ that identify interacting atom groups, evaluating the plausibility of interactions based on distances and angles. Interaction analyses often do not take into account water-mediated interactions, which are crucial to understanding the specificity and affinity of small molecule binding. Although certain tools^29^ have been developed, most lack simple integration into open-source MD pipelines.

We present *OpenMMDL*, a novel tool that extends *OpenMM*, with a particular focus on the efficient preparation of protein–ligand complexes for simulation. Furthermore, *OpenMMDL* incorporates integrated analysis tools for investigating molecular interactions and water kinetics. Designed with an emphasis on user-friendliness and extensibility, *OpenMMDL* is well-positioned to support a diverse array of applications in molecular dynamics, especially within the realm of computational drug design.

## Implementation

### *OpenMMDL Setup*

**Figure 1:**
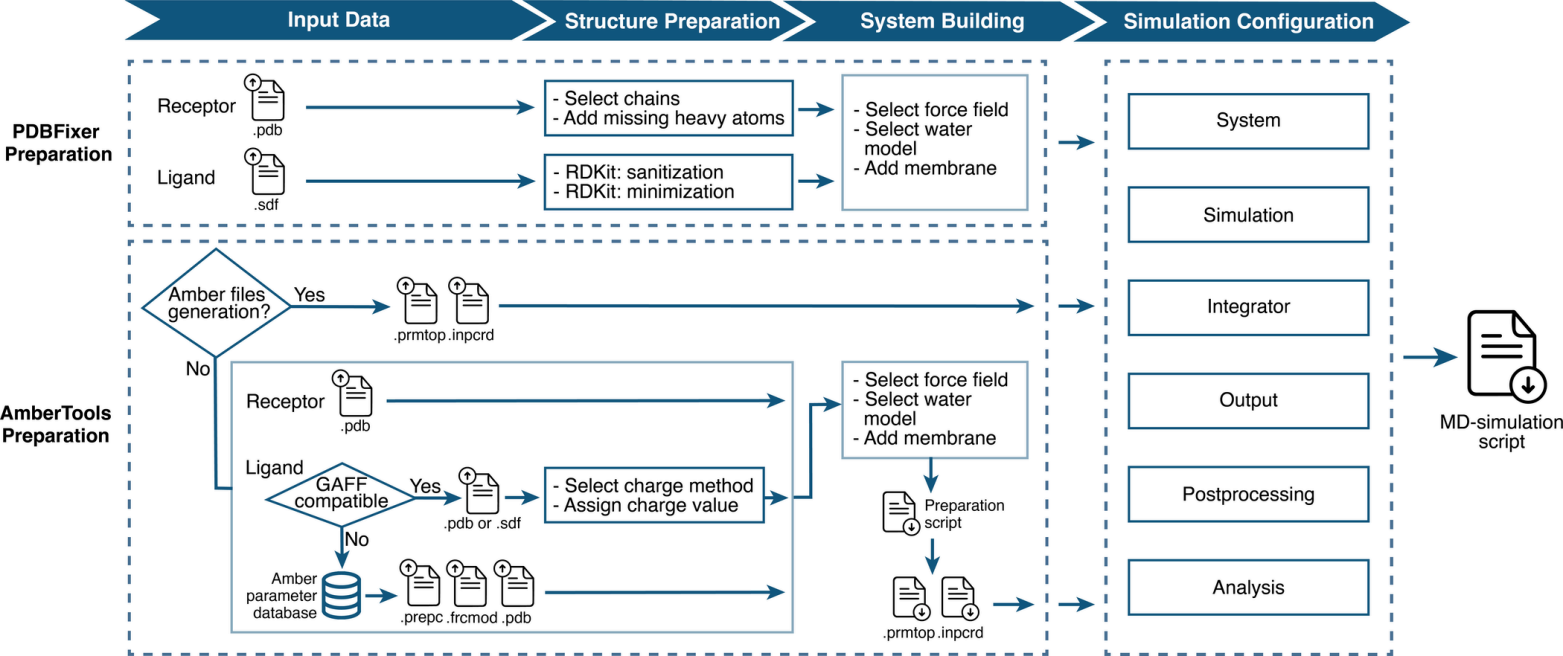
Schematic representation of simulation file preparation using *OpenMMDL Setup*. Two preparation options are featured: *PDBFixer Preparation* and *AmberTools Preparation*. The preparation process begins with file uploading. The *AmberTools Preparation* has the option to upload the *Amber* topology and coordinate files, which leads directly to the final page with the simulation configuration. With the option to upload PDB files, the subsequent steps involve preparing the structure, building the system, and configuring the simulation settings. This results in the delivery of an MD simulation script, which serves as input for *OpenMMDL Simulation*.

The *Flask*-based^30^*OpenMMDL Setup* provides access to a web-based GUI through any browser, offering two options for preparing an MD simulation. Simulations can be initiated either by directly using Amber input files or by starting with protein PDB and ligand SDF files, which can then be processed with *PDBFixer*(9) or *AmberTools*(4) (Figure 1).

#### *PDBFixer Preparation*

*OpenMMDL*’s *PDB Preparation* integrates *PDBFixer*,^9^ a package for PDB file preparation and *RDKit*,^10^ an open-source chemoinformatics toolkit applied in drug discovery and computational chemistry to allow system setup from PDB and SDF file inputs. *PDBFixer* addresses missing residues and heavy atoms, while *RDKit* is used for ligand minimization and sanitization. Subsequently, *OpenMMDL Setup* allows force field and water model selection, which are automatically applied to ensure seamless compatibility with *OpenMM*, along with water box and membrane customization.

#### *AmberTools Preparation*

The *AmberTools Preparation* generates *Amber* topology and coordinate files from separate ligand and protein PDB input structures,^4^ allowing to choose the force fields for both protein and ligand. *Antechamber*,^31^ designed to work with GAFF,^32^ plays a crucial role in the generation of ligand parameter files. Furthermore, the preparation enables the inclusion of GAFF-incompatible ligands, such as porphyrins. These ligand parameters are provided in the *Amber* parameter database.^33^ The system can also include a water box and a membrane, with the option of a membrane mixture with varying lipid ratios. This is achieved using *PACKMOL-Memgen*.^34^ A preparation script can be executed to generate topology and coordinate files, which serve as input for subsequent MD simulations.

#### Simulation Configuration

The simulation configuration determines simulation duration and frame count, as well as the output parameters that generate a data log file detailing specifics including the simulation progress. Checkpoint files, vital for restarts, are generated at a predefined 0.02 ns interval, and are modifiable. Postprocessing options include topology and trajectory file format selection. Output choices allow the definition of atoms to be included. The configuration also facilitates the execution of *OpenMMDL Analysis* on the final output with customizable default analysis parameters, along with offering options for simulation, integrator settings, and modification capabilities. Finally, a Python script is generated, which serves as the input for *OpenMMDL Simulation*.

### *OpenMMDL Simulation*

*OpenMMDL Simulation* facilitates the simulation of protein–ligand complexes from *OpenMMDL Setup*. The input supports PDB topology files with ligand files in SDF and MOL2 format. For the output of the *PDBFixer Preparation* module, *RDKit*(10) adds necessary hydrogens and edits chiral atoms, with optional energy minimization using the MMFF94 force field.^35−41^ The ligand is then converted to an *Open Force Field* Molecule^42^ and subsequently to an *OpenMM* object,^9^ to ensure coordinate correctness. *PDBFixer*(9) reads the protein topology and facilitates its merging with the ligand using *MDTraj*.^16^ The resulting complex is solvated using the specified water model and ions. Upon selection of padding options, a membrane is added accordingly. The output of the *AmberTools Preparation* utilizes the generated *Amber* topology and coordinate files, requiring no additional modifications. The simulation begins with an energy minimization step, followed by system equilibration and subsequent simulation.

#### Simulation Postprocessing

**Figure 2:**
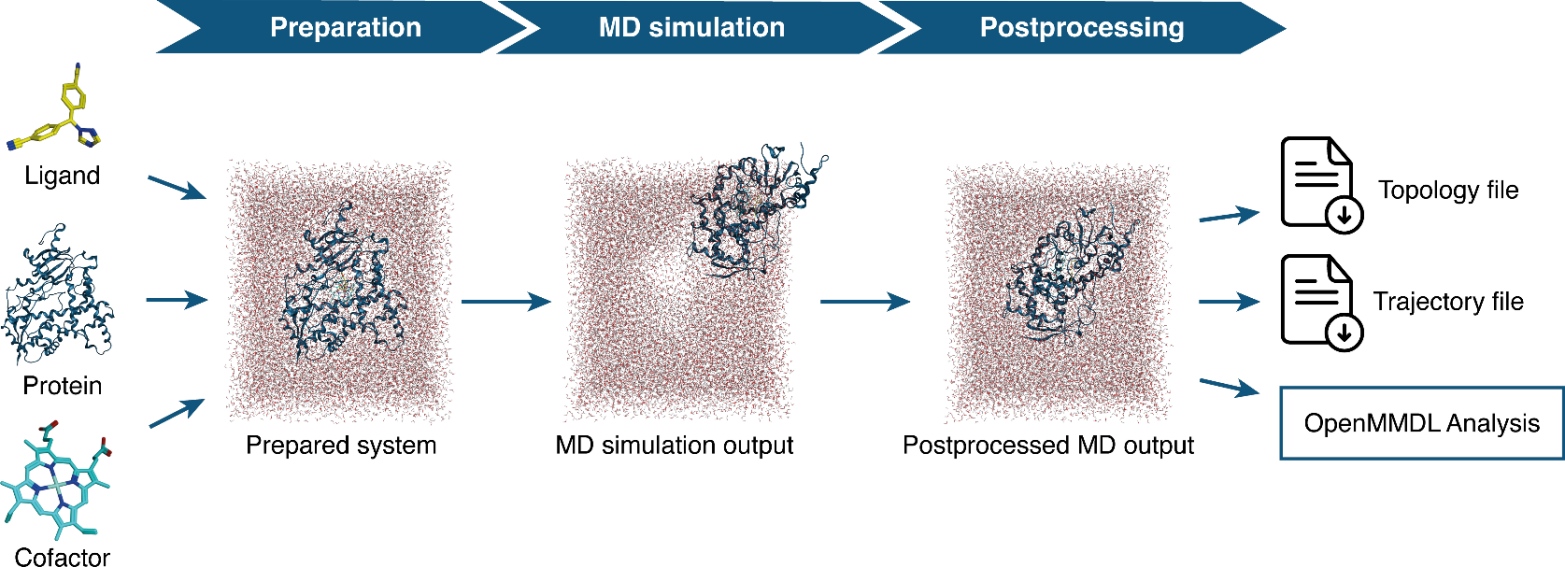
Simulation workflow overview. The simulation workflow involves combining the topologies of the protein, ligand, and cofactor to obtain the prepared system with the desired water model and membrane, if selected. The prepared system undergoes MD simulation to generate the MD simulation output. This output is then postprocessed to generate an aligned protein that is centered in the solvent with the initial coordinates.

Postprocessing is performed by *OpenMMDL Simulation*. The protein–ligand complex is centered inside the water box using *MDTraj*(16) and the complex and water box are repositioned to maintain the initial coordinates with *MDAnalysis*,^15^ both packages being used for MD simulation analysis. The final postprocessing step aligns the protein and ligand with the initial topology, preventing undesirable rotation. The final result is an analysis-ready trajectory (Figure 2).

### *OpenMMDL Analysis*

**Figure 3:**
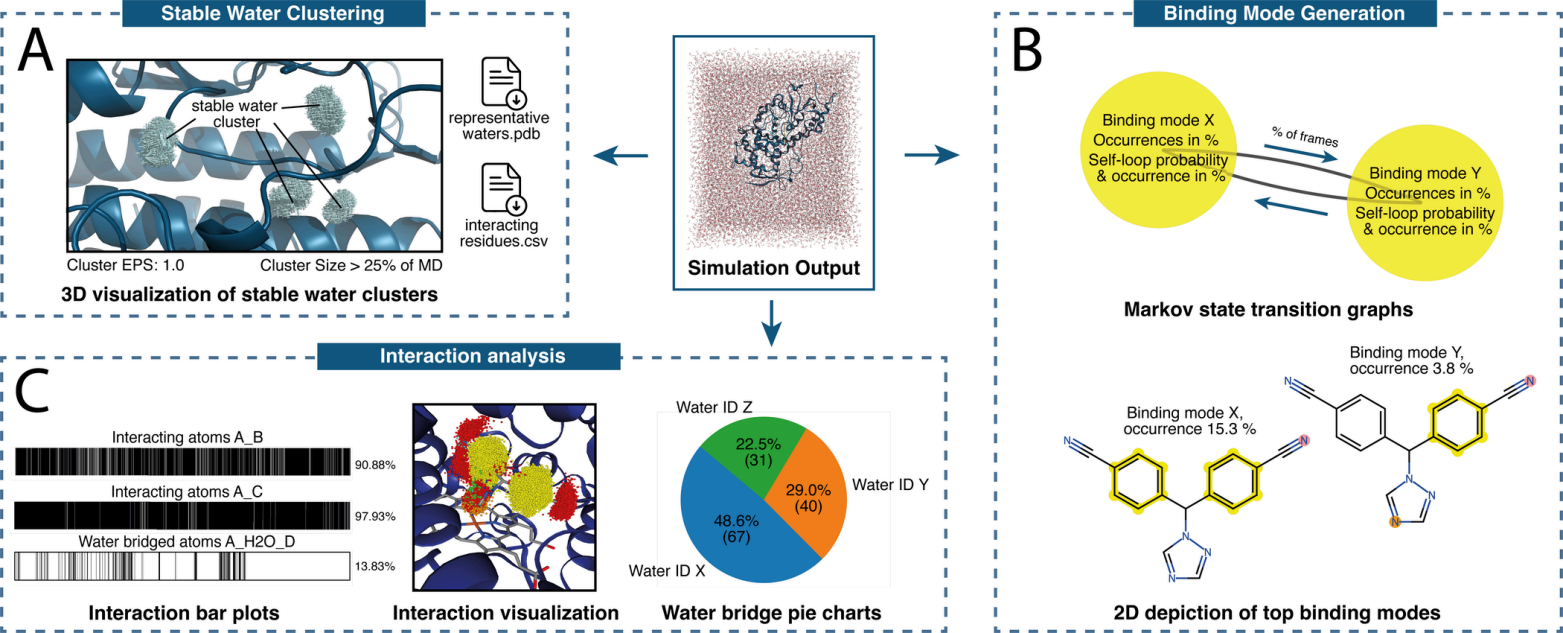
(A) The stable water analysis reveals clusters of water molecules that remain stable between two consecutive frames. The clusters in this example have a maximum distance of 1 Å (cluster EPS = 1.0) between each core member in at least 25% of the MD simulation. (B) The transition graph displays the individual binding modes, their occurrence frequency, and the transition percentage between them. The transition between binding modes is characterized using two values: the occurrence of the transition (transition/amount of frames) and the probability of the transition for the binding mode (transition/binding mode occurrence). Additionally, the self-loop probability of the binding mode, and thus the transition itself, is also displayed. (C) On the left, there is a “barcode” representation of interactions over the course of an MD trajectory. The chart on the right displays the total interaction frequency as a percentage. In the center, a point cloud representation of interactions over the course of the trajectory is shown. The colors represent different types of interactions: yellow for hydrophobic contacts, red for hydrogen bond acceptors, green for hydrogen bond donors, orange for metal interactions, dark blue for π-stacking, and cyan for water bridge interactions. The pie chart on the right shows the participation of three dominant water molecules in one water bridge found by the interaction analysis.

*OpenMMDL Analysis* allows users to obtain multiple analytical metrics from their MD trajectory. These metrics include root-mean-square deviation (RMSD) calculations of the protein backbone and ligand, as well as receptor–ligand interaction features. These are used to generate binding modes - fingerprints of interaction combinations in a certain frame. *OpenMMDL* also offers options for 2D and 3D visualization of molecular interactions. Additionally, *OpenMMDL* clusters stable waters, defined by a movement of less than 1 Å between consecutive frames (Figure 3).

The analysis core is based on the Python API of *PLIP*(18,19) and *MDAnalysis*.^15^ This allows the calculation of interactions within the protein–ligand complex and additional special ligands for each trajectory frame. Interaction data is processed using *Pandas*.^43^ This feature enables the creation of fingerprints that combine the most significant interactions in each frame, allowing *OpenMMDL Analysis* to identify various binding modes within the MD simulation trajectories. Furthermore, the transition between binding modes is tracked through Markov chains, which are generated using *NetworkX*.^44^ They show the probabilities of certain binding modes transitioning into others. The top 10 binding modes are depicted in 2D by occurrence. Interacting ligand atoms are highlighted with their corresponding interaction colors, displayed in the legend (Figure 3B). Additionally, a PDB file is created for a representative frame with the lowest RMSD to all other frames with the binding mode.

Occurrence plots are generated for each unique interaction between a ligand moiety and a specific receptor residue. Additionally, grouped occurrence plots for each unique interaction type formed by a portion of the ligand are created. Each barcode section represents a distinct frame. The presence or absence of an interaction is indicated by the black or white color, respectively.

Point clouds, an alternative method of interpreting interactions, have been instrumental in studies using *Dynophores* (in *LigandScout*).^20−25^*OpenMMDL* generates a point cloud with a single point representing each occurrence of an interaction in a given frame. The point’s position is determined by the ligand moiety’s coordinates involved in the interaction being represented. The interaction clouds can be viewed alongside the trajectory in a prepared *Jupyter Notebook* using *NGLview*.^45^

Protein–ligand interaction analysis is deepened through the characterization of water-mediated interactions and water tracing, which examines the movement of water molecules between consecutive frames. Additional barcodes are assigned for water-bridged interactions, and pie charts display specific waters involved in each water bridge. (Figure 3C). Water tracing collects water molecules showing under 1 Å of movement and uses Density-Based Spatial Clustering of Applications with Noise (*DBSCAN*)^46^ to identify clusters of low water movement. *DBSCAN* is limited by the ability to detect clusters of varying densities, and its dependence on input parameters,^47^ as it is very sensitive to changes in these parameters.^48^ Therefore, the distance parameter for *DBSCAN* can be set during setup, with the initial chosen default set to 1 Å. This ensures that clusters contain only one water molecule, as water molecules are generally at least 3 Å apart from each other.^49^ As clustering occurs within a simulation with similar parameters, the density of a desired stable water cluster is comparable, which makes *DBSCAN* a suitable approach. To ensure reasonable clustering in different experimental setups, the clustering output comprises five folders by default, containing clusters of different sizes (stable waters present in 25%, 50%, 75%, 90% and 99% of the simulation), allowing clusters to be sorted by their amount of occurrence during the simulation. The clusters highlight the regions where water molecules interact with the protein–ligand complex or are trapped (Figure 3A). For each cluster, representative water molecules and a list of potentially participating protein residues are retrieved.

## Methods

### Structure Preparation

Experimental structures were obtained from RCSB PDB^50^ and OPM database.^51^ The structures were prepared using *MOE 2022.02*.^52^ Missing loops were modeled with the *Loop Modeler* tool, and the structures were subsequently prepared with *Structure Preparation*. Chain breaks were capped with ACE and NME. The structure was protonated using *Protonate 3D* at pH 7.0 and a temperature of 300 K. Nonmembrane proteins were solvated in an orthorhombic box containing TIP3P water, 0.15 M NaCl, and 10 Å padding. Membrane proteins were embedded in a POPC lipid bilayer, as positioned in the OPM database,^51^ with TIP3P water, 0.15 M NaCl, and a 10 Å padding.

### MD Simulation

MD simulations were performed on RTX4090, RTX3090 and RTX2080Ti GPUs (NVIDIA Corporation, Santa Clara). *PDBFixer Preparation* systems were simulated using the Amber ff14SB force field,^53^ while *AmberTools Preparation* systems utilized the Amber ff19SB force field.^54^ Long-range electrostatic interactions were calculated using the Particle Mesh Ewald method with a 10 Å cutoff. The length of all bonds involving a hydrogen atom was constrained. A Monte Carlo barostat was applied to the system with a pressure of 1 bar and a temperature of 300 K. The simulation was performed using Langevin dynamics at 300 K, a friction coefficient of 1 ps^–1^, and a time step of 2 fs. Ten replicas of 100 ns were generated using an NPT ensemble and periodic boundary box conditions. The simulation coordinates were saved every 100 ps, generating 1000 frames for each trajectory.

### MD Analysis

Trajectories were concatenated in *VMD*.^13^ The exceptions were the case studies on the 5-HT_2B_ receptor and the Ca_*v*_1.1 channel, which were inspected individually. This resulted in single trajectories of 10,000 frames that were used for analysis. The MD simulations were analyzed using *OpenMMDL Analysis* with the binding mode threshold set to the default value of 40%.

### Coding and Writing

*ChatGPT* [OpenAI, L.L.C., San Francisco, CA] as well as *DeepL Write* [DeepL SE, Cologne, Germany] were used in the writing process for spelling and grammar corrections. *ChatGPT* [OpenAI, L.L.C., San Francisco, CA] and *GitHub Copilot* [GitHub Inc., San Francisco, CA] were used for programming assistance throughout the development of *OpenMMDL*.

## Case Studies

### *PDBFixer Preparation* Case Studies

#### ALK2 Kinase Bound to LDN-212854 and LDN-193189

Kinases, such as activin-like-kinase 2 (ALK2), have great pharmaceutical relevance due to their role in various diseases and cellular processes.^55^ However, high structural similarity within the kinome renders the difficult design of kinase-specific compounds, often leading to off-target effects. Therefore, focusing on the water molecule network is crucial for effective drug design and structure–activity relationship studies.^56−58^ This is exemplified by the selectivity of LDN-212854 over LDN-193189 for ALK2, even though the binding modes of both compounds within the active site of ALK2 are nearly identical.^58^ The nitrogen in the 5-quinoline of LDN-212854 was proposed to form a water-mediated hydrogen bond with both K235 and E248 of ALK2.^59^ LDN-193189 also interacts with E248 via a water molecule, but this water is displaced by 1.54 Å, out of reach for interaction with K235.^58^

**Figure 4:**
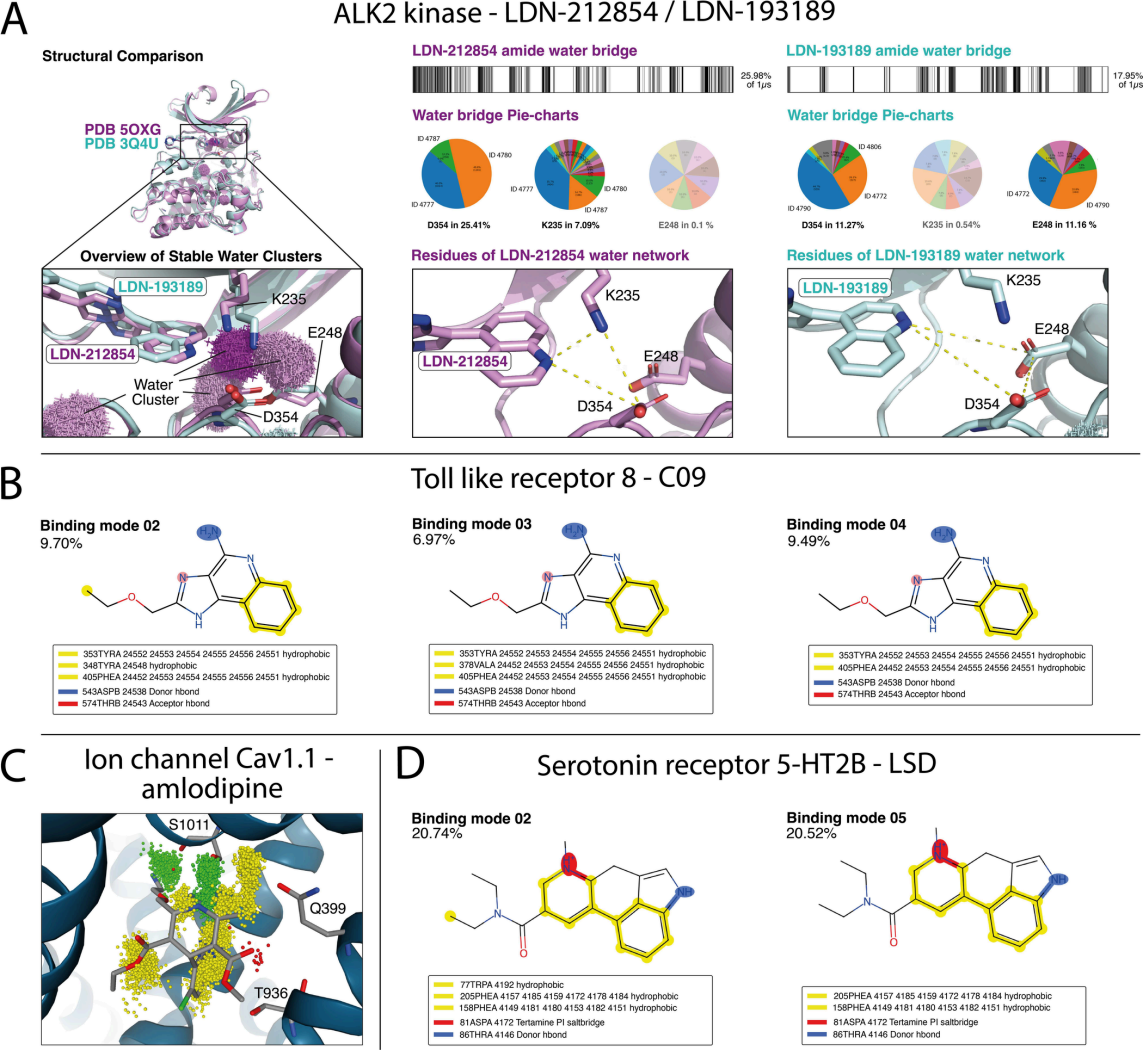
*OpenMMDL Analysis* of simulated systems prepared with the *PDBFixer Preparation*. (A) Comparison of the BMP receptor kinase ALK2 bound to the compounds LDN-212854 (magenta) and LDN-193189 (cyan). Simulating LDN-212854 in ALK2 resulted in three stable water clusters at the binding site (cluster EPS: 1 Å; 25% of 1 μs simulation). On the right, water bridge analysis shows LDN-212854 forming bridges in 26% of the MD (predominantly with D354 and K235) and LDN-193189 in 18% (with D354 and E248), with pie charts indicating the involved water molecules. (B) Study of TLR8 binding the ligand C09: top three occurring binding modes of C09 during a 1 μs MD simulation are shown. Binding mode 2 displays an additional interaction with Y348, and binding mode 3 displays an interaction with F405. Binding mode 4 is a transitional binding mode between binding modes 2 and 3. (C) Interactions seen throughout one simulation replicate (100 ns) of amlodipine in Ca_*v*_1.1, with hydrophobic contacts (yellow point clouds), hydrogen bond donors (green point clouds), and hydrogen bond acceptors (red point clouds). (D) Binding modes 2 and 5, representing the two distinct binding modes that LSD can occupy in the 5-HT_2B_ receptor.

Using *OpenMMDL Analysis* to analyze the ALK2-inhibitor complexes (PDB ID: 3Q4U and^60^ 5OXG),^58^ we discovered that only LDN-212854 forms stable water clusters at the interaction site in over 25% of the simulation (shown in Figure 4A, left). LDN-212854 forms water bridges in 26% of the simulations, compared to the 18% shown by LDN-189193. Pie chart visualization of water bridges indicates that the K235 of ALK2 interacts with LDN-212854, but not LDN-193189, in agreement with the literature.^58^ Surprisingly, E248 of ALK2 forms water bridges with LDN-193189 in 11.17% of MD simulations, but not with LDN-212854, in contrast to the prediction from static X-ray crystal structure analyses.^58,59^ Instead, LDN-212854 forms a water bridge toward D354 in 25.41% of MD simulations. This water bridge is formed by three water molecules, suggesting high stability (Figure 4A, right). Interestingly, 4-sulfamoylnaphthyl, a nanomolar ALK2 inhibitor, directly interacts with D354 and K235.^59^ Its sulfonamide group may replace the water bridge we identified between LDN-212854 and D354/K235, which strengthens the finding about the relevance of this water bridge for ALK2 selectivity.

#### Toll-like Receptor 8 Bound to Ligand C09

Toll-like receptors (TLRs) are a family of pattern-recognition receptors, which are responsible for recognizing the pathogen-associated molecular patterns (PAMPs) of bacteria, viruses, parasites and fungi. Thus, they play an important role in the innate immune system,^61,62^ and TLR8 agonists have been studied for their potential role in treating cancer, asthma, viral and bacterial infections.^62^ We used *OpenMMDL* to simulate and analyze the structure of a TLR8 agonist C09 (PDB ID: 3W3J).^63^

The main interactions important for TLR8 receptor agonism that are featured in the X-ray crystal structure of the TLR8 agonist C09 are the hydrogen bond interactions with D543 and T574 in addition to hydrophobic contacts with residues F346 Y348, Y353, V378, I403, F405 and V573. *OpenMMDL Analysis* used a binding mode threshold of 35%, which was necessary to include the hydrogen bonding interaction with D543. The two most frequently occurring binding modes during the simulation, binding modes 2 and 3, both exhibit hydrogen bond interactions with D543 and Y574, and hydrophobic contacts between the phenyl ring of C09 and the residues F405 and Y353. Binding mode 2 features an additional hydrophobic contact between the alkyl chain of C09 and Y348, while binding mode 3 displays a contact between the phenyl ring and V378 instead. The third most frequent, binding mode 4, is a transitional binding mode, which lacks both V378 and Y348 interactions, but still exhibits interactions with Y353, F405, D543, and T574 (Figure 4B). This case study demonstrates the application of OpenMMDL for simulating multimers and showcasing ligand binding modes, including transitional binding modes that occur during the transition between more stable binding modes.

#### Voltage-Gated Calcium Channel Ca_*v*_1.1 Bound to Amlodipine

Voltage-gated calcium channels (Ca_*v*_) are membrane-bound proteins that mediate Ca^2+^ influx.^64^ The Ca_*v*_1.1 subtype, specifically expressed in skeletal muscles, triggers muscle contraction upon depolarization.^65^

A well-studied class of Ca_*v*_1.1 antagonists are the 1,4-dihydropyridines (DHPs), primarily developed for the treatment of cardiovascular diseases,^66^ such as nifedipine and amlodipine, both of which have been captured in complex with Ca_*v*_1.1 by cryo-EM.^67,68^ Both bind close to the pore of the α_1_-subunit to a hydrogen bond between the hydroxy oxygen of S1011 in Ca_*v*_1.1 and the N1-nitrogen of the DHP-ring. Mutation of S1011 significantly reduces activity.^69^ Additionally, amlodipine contains an ethanolamine chain that extends into the Ca_*v*_1.1 pore and forms a hydrogen bond to the carbonyl oxygen of S1011.^68^

The Ca_*v*_1.1 α_1_-subunit-amlodipine complex (PDB ID: 7JPX) was simulated and visually inspected (Figure 4C). The protein and ligand remained in the complex throughout all replicas. Eight out of ten replicas show the formation of the expected hydrogen bonds between the DHP-nitrogen and the ethanolamine chain with S1011. Among these eight replicas, amlodipine displays an average RMSD of 2.26 Å. However, the other two replicas show amlodipine assuming a different binding mode without the hydrogen bond between S1011 and the DHP-nitrogen. The RMSDs of amlodipine in these two replicas are 3.55 and 4.10 Å.

#### Serotonin Receptor 5-HT_2B_ Bound to LSD

The 5-HT_2B_ receptor, a member of the G-protein coupled receptor family (GPCRs), binds the endogenous ligand serotonin. The selective shift of the conformational equilibrium by GPCR ligand binding modulates downstream signaling pathways mediated by arrestins and G proteins.^70,71^ The three Cryo-EM structures of 5-HT_2B_7SRQ, 7SRR and 7SRS(72) describe the receptor in a partially active, transducer-free state as well as two fully active states that are G_*q*_-protein and β-arrestin-1 coupled, respectively. As arrestins and G-proteins bind the same cytoplasmic interhelical cavity, and their affinities depend on the phosphorylation state of the GPCR and the presence of GTP,^73^ a detailed understanding would facilitate the development of future drugs with greater efficacy and fewer adverse effects.^74^ Thus, we analyzed possible binding modes of LSD in the transducer-free state.

We compared our results to the previously published binding mode of LSD in 5-HT_2B_,^75^ as well as the X-ray crystal structure of LSD bound to 5-HT_2B_ (PDB ID: 5TVN).^76^ Our results indicate several possible binding modes for LSD in 5-HT_2B_, two of which are depicted in Figure 4D. The main difference lies in the lipophilic contacts: the two ethyl groups of the diethylamide moiety, which are freely rotatable, interact with L78^3.29^, W77^3.28^, L227^7.35^ and V231^7.39^, as well as L150^*ECL2*^. These residues are part of the extended binding pocket and, depending on the binding mode, they are displayed in different combinations. This is of particular interest since specific diethylamide configurations are proven determinants for LSD’s β-arrestin signaling profile and ligand-specific receptor functions.^75^

The salt bridge between D81^3.32^ and the basic nitrogen of the ergoline ring of LSD, mandatory for the activation of aminergic receptors, appeared in every frame of the trajectory. Additionally, we observe a π-stacking aromatic interaction between the indole moiety and F206^6.51^. However, the hydrogen bond between the indole nitrogen and G162^5.42^ is only present in 12% of the frames. This may be due to the restrictive default settings for hydrogen bonds in *PLIP*.^18,19^

### *AmberTools Preparation* Case Studies

#### Cytochrome P450 Enzyme CYP19A1 Bound to Letrozole

**Figure 5:**
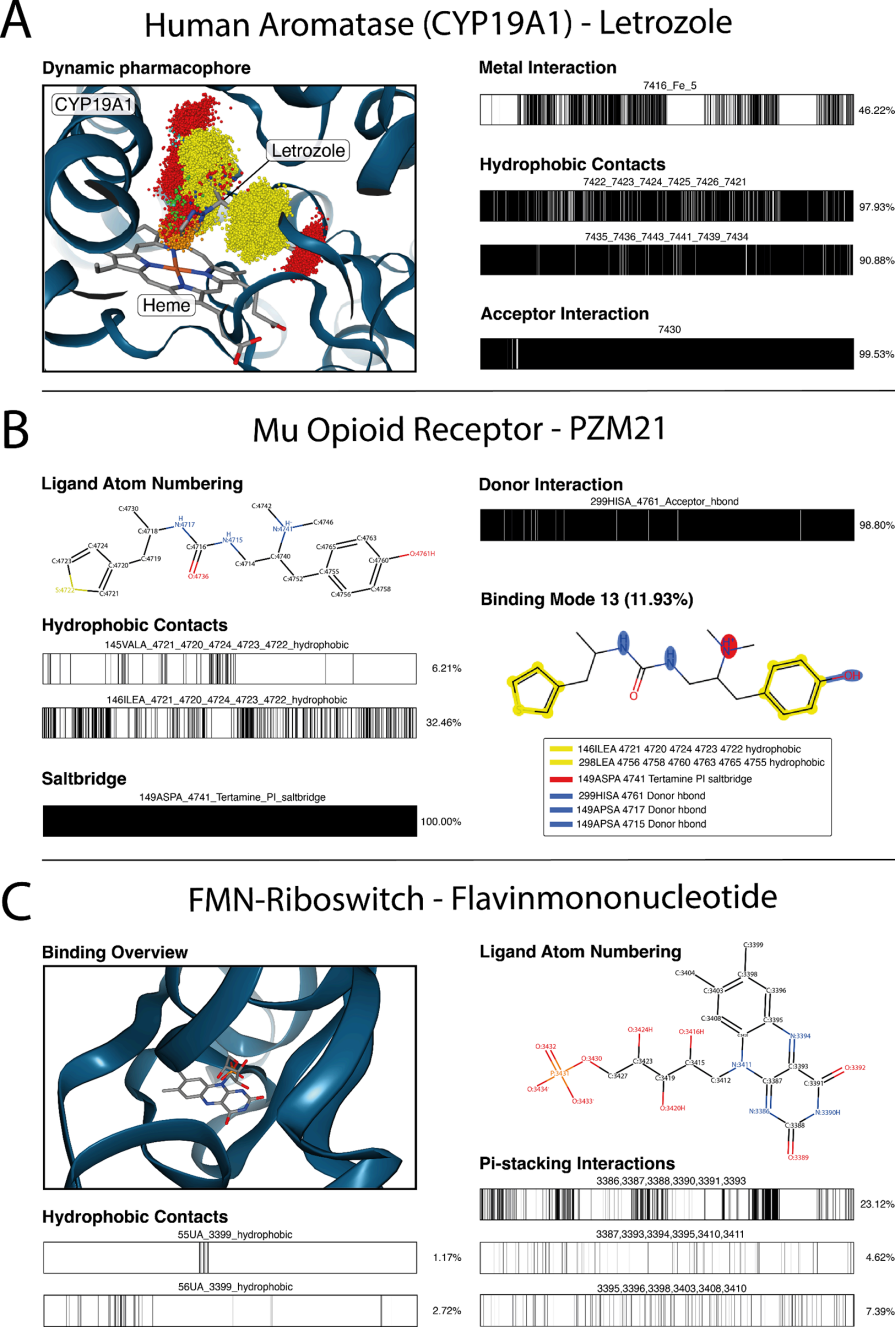
*OpenMMDL Analysis* results of systems simulated with the *AmberTools Preparation*. (A) Interaction clouds and occurrence plots for tracing the interactions between letrozole and CYP19A1 with Heme. (B) Study of μ-opioid receptor binding the ligand PZM21. Ligand atom numbering of PZM21, the occurrence plots for interactions between PZM21 and key residues in MOR over the MD trajectories, as well as the most occurring binding mode. (C) 3D and 2D representation of FMN and interaction occurrence of π-stacking and hydrophobic interactions over the course of the MD trajectory.

Aromatase, or CYP19A1, is a cytochrome P450 (CYP) enzyme with a crucial role in estrogen biosynthesis.^77^ Aromatase has long been a clinical target in the treatment of estrogen receptor-positive (ER+) breast cancer, with aromatase inhibitors (AIs) being a cornerstone in therapeutic approaches.^78^ Letrozole is a prominent third-generation nonsteroidal AI,^78^ and understanding its CYP19A1 inhibition is crucial for developing novel breast cancer treatments. We performed molecular docking of letrozole into the binding site of aromatase to generate a starting ligand-protein complex, paying attention to the accurate recognition, implementation, and simulation of the special ligand *Heme*, which is the prosthetic group of the P450 family. We used *OpenMMDL Analysis* to trace the interaction frequency between the Heme iron and the nitrogen lone electron pair of the triazole group throughout the MD simulations. We evaluated the binding stability of letrozole to CYP19A1 through interaction clouds and barcode analysis (Figure 5A). The metal-coordinating interaction feature (from atom N7416) was present in seven of the ten distinct binding modes, indicating a consistent interaction between letrozole and aromatase Heme. The detected interaction features of the binding modes include lipophilic contacts formed by the phenyl rings, mainly to neighboring residues F90, V326, and F177. Additionally, a hydrogen bond is formed from the nitrile group to M330 (acting as a donor) in all binding modes.

#### μ-Opioid Receptor Bound to Biased Ligand PZM21

The μ-opioid receptor (MOR) is the primary receptor for opioid analgesics, and therefore also their associated side effects.^79^ Upon activation of MOR, signaling pathways are initiated through both G proteins and the recruitment of β-arrestin, and a hypothesis gaining a lot of attention in the opioid receptor (OR) community suggests that G-protein-biased MOR agonists may exhibit diminished unwanted effects.^80^ In a study driven by this hypothesis, computational docking followed by structure-based optimization led to the identification of PZM21, which demonstrated reduced side effects in mice.^81^ Despite recent challenges to this hypothesis,^82−84^ substantial efforts have been directed toward resolving the structures of PZM21 bound to MOR,^85,86^ as these structural insights provide a foundation for understanding the structural basis of G-protein biased agonism. This case study focused on the experimentally solved structure of PZM21 bound to the human MOR (PDB ID: 8EFO)^86^ as a model to illustrate the application of *OpenMMDL Setup* with *AmberTools Preparation* for membrane-embedded system setup. Subsequently, *OpenMMDL* Analysis was employed to analyze key interactions.

The original structure, obtained from the OPM database for proper membrane placement using *PACKMOL-Memgen*, was prepared after conducting structure preparation in MOE. Notably, the dimethylamino moiety of PZM21 was recognized as charged at pH 7.4. The prepared structure was then used by the *AmberTools Preparation* within *OpenMMDL Setup*. During *OpenMMDL Analysis* with default settings, challenges were encountered in correctly converting the charged PZM21 to an SDF file recognizable by *OpenMMDL Analysis* using *OpenBabel*. An SDF file of PZM21 was prepared in *MOE* instead and then specified using the ‘-l’ flag in *OpenMMDL Analysis*. To ensure accurate residue numbering adhering to the Ballesteros–Weinstein system, the ‘-ref’ flag was employed to align the residue numbers in the output topology file of the MD simulation with the reference protein.

Throughout the trajectory, the positive nitrogen of PZM21 maintained a persistent polar interaction with D149^3.32^ (100.00% of the MD trajectory) (Figure 5B, lower left). This interaction is universally observed in opioid receptors and is crucial for MOR activation.^87−90^ The phenolic moiety of PZM21 that is initially oriented toward transmembrane helix 5 (TM5) in the starting structure gradually shifted to form a hydrogen bond with H299^6.52^ (98.80%) (Figure 5B, upper right). This interaction is crucial for both G protein and β-arrestin signaling activities.^86^ The thiophene moiety of PZM21 exhibits hydrophobic contacts similar to those seen with neutral agonists including fentanyl.^86^ However, it is noteworthy that these interactions occurred less frequently during the MD simulation (V145^3.28^ - 6.21%, I146^3.29^ - 32.46%) (Figure 5B, middle left). PZM21 is hypothesized to exhibit reduced arrestin activity because it forms fewer interactions with TM6/7. Specifically, it lacks close contacts with W295^6.48^, G327^7.42^, and Y328^7.43^, compared to TRV130. Despite being a biased agonist, TRV130 still has weak arrestin activity.^86^ The occurrence plots of the benzene ring of the phenol moiety show either no or very low frequencies of the aforementioned interactions during the MD trajectory, supporting the hypothesis regarding the interaction feature pattern TM6/7. The most common binding mode 13, displays all necessary interactions for the G-biased agonism of PZM21 (Figure 5B, lower right).

#### FMN-Riboswitch Bound to Flavin Mononucleotide

Riboswitches are regulatory elements found in mRNA (mRNA) that sense various metabolites and ions to regulate gene expression. Flavin mononucleotide (FMN) riboswitches are a promising target for broad-spectrum antibacterial drug development due to their prevalence in a wide range of bacterial species.^91−94^ We used the X-ray crystal structure of the FMN riboswitch bound to FMN (PDB ID: 3F2Q)^95^ to demonstrate the competence of OpenMMDL in simulating and analyzing RNA-ligand complexes (Figure 5C). The system was prepared by deleting the modified triphosphate tips of the two RNA chains and protonating the system with Protonate3D in *MOE*. The cocrystallized ions were retained, especially magnesium ions, which have been shown to stabilize RNA structures.^96^ The *AmberTools Preparation* enabled easy application of suitable AMBER force field parameters to each part of our system.

*OpenMMDL Analysis* enables us to monitor the key interactions of ligands with RNA receptors using the “-nuc True” flag. Since nucleic acids are highly polar systems, ligand binding is strongly directed by hydrogen bond interactions. In the case of FMN, we observe an abundance of hydrogen bonds being formed throughout the MD trajectory. Additionally, Figure 5C shows the π-stacking interactions between the nucleic acid bases and FMN, which further strengthen the binding of the ligand to RNA molecules; the RNA bases are stacked neatly upon each other, allowing for a significant energy gain when a ligand intercalates between them. However, the occurrence of π-stacking in the analysis is less frequent than expected. This may be due to insufficient interaction recognition through *PLIP*(18,19) in some cases. To improve the recognition of specific interactions, manual editing of the tolerances in the *PLIP*(18,19) configuration files may be necessary. However, in this case, we did not edit the recognition thresholds in order to maintain the reproducibility of the results. Finally, we identified hydrophobic contacts with the RNA, as shown in Figure 5C. It is uncommon for polar nucleic acids to form these contacts, yet information on these rare interaction features could prove invaluable in the design of novel ligands.

## Discussion

Despite *OpenMMDL*’s versatility, discussing its limitations is crucial for further development and usage. While the integration of *PDBFixer* provides basic functionalities for protein cleanup, it may have difficulty handling cocrystallized ligands. To address this, it is recommended to use modeling tools such as *MOE*(52) or *Maestro*.^97^*OpenMMDL* cannot construct systems with covalently bound ligands, as these rely on the GAFF force field^32^ along with expertise and careful validation of the user. However, *OpenMMDL* allows for the inclusion of special ligands incompatible with GAFF, such as the Heme in the CYP19A1 case study, via the *Amber parameter database*.^33^

During *PDBFixer Preparation*, users can provide ligand input in SDF or MOL2 format. The SDF format is recommended, with manual review prior to input to ensure chemically accurate structures, including proper assignment of bond orders for aromatic rings. Optionally, *RDKit*(10) can be used in this step for ligand minimization and sanitization, performing checks such as valence validation, aromaticity detection, conjugation, and hybridization assignment. Problems can arise in this automated process with nonstandard ligands, such as those with delocalized charges or unusual valence patterns. For example, we encountered a compound with a thiophene moiety that could not be sanitized, likely due to a failure to assign double bonds to the aromatic ring. It is also reported that some ligands with improper valences, such as a nitrogen atom with four bonds that lacks a formal positive charge, and some ligands with atoms with specific bonds to a metal ion, lead to sanitization errors in *RDKit*.^10^ In the latter case, an alternative approach is to use the *AmberTools Preparation* in the *OpenMMDL Setup*, where the nonstandard ligand can be properly prepared using the Amber Parameter Database. Careful evaluation of the simulation system’s PDB file and the final trajectories is crucial to ensure accurate representation and behavior.

Selecting water and membrane parameters with *PDBFixer* may result in inconsistencies in the number of water atoms across different system builds, although identical atoms in each replica are necessary for post-MD analysis. Fortunately, *AmberTools Preparation* provides a reliable solution to address the problem of atom inconsistency through *PACKMOL-Memgen*, for which it is necessary to align the entire structure with the corresponding OPM structure.^51^

The standardized MD protocol provided by *OpenMMDL* covers minimization, equilibration, and production runs, but experienced users should customize the protocols to their specific needs.

*OpenMMDL* can be used to postprocess MD trajectories for analysis with *MDTraj*(16) and *MDAnalysis*.^15^ However, these tools may encounter difficulties when handling systems with more than 9999 atoms. In cases such as the Ca_*v*_1.1 ion channel, which features a large membrane, the solution has been to remove the membrane and water components to enable postprocessing within this constraint. To this end, *OpenMMDL Analysis* offers a useful postprocessing function: residue renumbering. This is necessary because when the original protein PDB file is input into *AmberTools* or *PDBFixer*, the residue numbering restarts from 1, leading to inconsistencies between the original PDB file and the output system. Maintaining the original residue numbering is crucial for accurately identifying interactions with specific residues, especially in the case of GPCRs such as 5-HT_2B_ and MOR, which follow the Ballesteros-Weinstein numbering convention.^98^ To address this issue, OpenMMDL Analysis provides the ‘-ref’ flag, which aligns residue numbers in the output topology file with the reference protein.

When creating 2D representations of molecular interactions using *RDKit*(10) in postprocessing, it is important to exercise caution. *OpenMMDL Analysis* can automatically generate a ligand SDF file from the system’s topology file using *OpenBabel*.^99^ However, as shown in the MOR case study with PZM21, this method may not always produce an SDF file that can be recognized by *RDKit*,^10^ which could result in errors. Therefore, using the ‘-l’ flag and providing an SDF file for the ligand that was prepared beforehand is recommended.

*OpenMMDL Analysis* provides time series interaction analysis in the form of bar codes and point cloud visualization. These techniques draw on the methodology initially outlined by the *Dynophore App*(20−25) tool, built upon the *LigandScout*(26) structure-based 3D pharmacophore generation algorithm. This representation allows users to visualize interactions throughout the simulation. One current drawback in the *OpenMMDL Analysis* implementation concerns the underlying interaction identification algorithm. *OpenMMDL Analysis* uses *PLIP*,^18,19^ which sees less utilization compared to the *LigandScout*(26) 3D pharmacophore generation algorithm, due to issues such as the π-stacking example in the FMN case study. *OpenMMDL Analysis* offers an advantage over *Dynophore App* in its current state, due to being free for use and open-source.

## Conclusion

This study presents *OpenMMDL*, an open-source tool for preparing protein–ligand complexes for MD simulations using *OpenMMDL Setup*, performing these MD simulations with *OpenMM* through *OpenMMDL Simulation*, and analyzing the MD simulation trajectories to display the protein–ligand interactions and stable waters with *OpenMMDL Analysis*. The tool offers low-code users a straightforward way to access and fully use MD simulations. *OpenMMDL* was applied to various pharmaceutical drug targets, demonstrating its potential applications in modern drug discovery.

## Data and Software Availability

OpenMMDL is open-source, and the code is freely available at https://github.com/wolberlab/OpenMMDL. Documentation and tutorials for OpenMMDL are available at https://openmmdl.readthedocs.io.
